# Conference Calendar

**DOI:** 10.1002/cfg.162

**Published:** 2002-04


					Conference Calendar
June-August 2002
Animal models
13th
Intl. Workshop on the Molecular and
Developmental Biology of Drosophila - June
23-29
http://flybase.bio.indiana.edu : 82/docs/news/
announcements/meetings/CreteXIII.html
Mouse Initiatives IV - July 31-Aug 3
http://www.jax.org/courses/documents/mouse_initiatives_
02.html
9th
International Xenopus Conference - Aug
21-25
http://www.welc.cam.ac.uk/xenoconf/
CSHL - Mouse Molecular Genetics - Aug
28-Sept 1
http://nucleus.cshl.org/meetings/2002mouse.htm
Bacteria
7th Symposium on Bacterial Genetics and
Ecology - June 15-19
http://www.im.uib.no/nyheter/Bageco7/
CSHL - Molecular Genetics of Bacteria &
Phages - Aug 20-25
http://nucleus.cshl.org/meetings/2002phage.htm
Bioinformatics
Theoretical Biology & Biomathematics - June
9-14, 2002
http://www.grc.uri.edu/programs/2002/theobio.htm
ISMB 2002 (Intelligent Systems in Molecular
Biology) - Aug 3-7
http://www.ismb.org/ismb2002
Evolution/comparative genomics
Molecular Evolution 2002 - June 13-16
http://www.szn.it/molevol02/
Genomics & Structural/Evolutionary
Bioinformatics - July 28-Aug 2
http://www.grc.uri.edu/programs/2002/genom.htm
General genomics
CHI - Beyond Genome (In Silico Biology,
Bioinformatics, Proteomics) - June 2-7
http://www.beyondgenome.com/
Metabolomics
GRC - Enzymes, Coenzymes & Metabolic
Pathways - July 21-26
http://www.grc.uri.edu/programs/2002/enzymes.htm
Pharmacogenomics
Intl. Conf. on Genomics, Proteomics and
Bioinformatics for Medicine - June 22-29
http://www.ibmh.msk.su/gpbm2002/
Plants
1st
Intl Conference on Legume Genomics and
Genetics - June 2-6
http://www.agro.agri.umn.edu/iclgg/
Comparative and Functional Genomics
Comp Funct Genom 2002; 3: 209-210.
Published online 14 March 2002 in Wiley InterScience (www.interscience.wiley.com). DOI: 10.1002/cfg.162
Copyright # 2002 John Wiley & Sons, Ltd.
3rd Intl Cotton Genome Initiative (ICGI)
Workshop - June 3-6
http://icgi.tamu.edu/nanjing/
International FAO/IAEA Symposium on the
Use of Mutated Genes in Crop Improvement
and Functional Genomics - June 3-7
http://www.iaea.or.at/worldatom/Meetings/2002/
infcn89.shtml
13th Intl Conference on Arabidopsis Research
- June 28-July 2
http://www.arabidopsis2002.com/
GRC - Plant Molecular Biology - July 7-12
http://www.grc.uri.edu/programs/2002/plantmol.htm
Proteomics
50th ASMS Conference on Mass Spectrometry
- June 2-6
http://www.asms.org/confASMS2002Prelim.php
16th Symposium Of The Protein Society - Aug
17-21
http://www.faseb.org/meetings/ps02/index.html
5th Intl. Symposium on Mass Spectrometry in
the Life Sciences - Aug 26-30
http://donatello.ucsf.edu/symposium/
Structural genomics
GRC - Diffraction Methods In Structural
Biology - July 14-19
http://www.grc.uri.edu/programs/2002/diffrac.htm
Transcriptomics
EuroBioChips 2002 - June 25-28
http://www.eurobiochips.com/euro2002/html/pre-
introduction.asp
Yeasts and fungi
EURESCO conference: Gene Transcription in
Yeast - 31 May-5 June
http://www.esf.org/esf_euresco_conference.php?
conference=103&meeting=4
6th Intl Meeting on Molecular Epidemiology
and Evolutionary Genetics in Infectious
Diseases (MEEGID - VI) - 24-27 July
http://viradium.mpl.ird.fr/meegid6/index2.html
Yeast Genetics and Molecular Biology Meeting
- July 30-Aug 4
http://www.faseb.org/genetics/yeast/
Zebrafish
Fifth International Conference on Zebrafish
Development & Genetics - June 12-16
http://zfin.org/zf_info/news/mtgs.html#Madison
Biotech industry
Bio2002 - June 9-12
http://www.bio.org/events/2002/index.htm
9th
Annual Euro-Biotech Forum - June 23-25
http://www.windhover.net/conf/eb/9ebweb02.asp
Key
ASMS - American Society for Mass Spectrometry:
http://www.asms.org/
CHI - Cambridge Healthtech Institute: http://
www.healthtech.com/
CSHL - Cold Spring Harbor Laboratory: http://
www.cshl.org/
FAO - Food and Agriculture Organization of the
United Nations: http://www.fao.org/
GRC - Gordon Research Conference: http://
www.grc.uri.edu/
IAEA - International Atomic Energy Agency:
http://www.iaea.org/worldatom/
210 Conference Calendar
Copyright # 2002 John Wiley & Sons, Ltd. Comp Funct Genom 2002; 3: 209-210.

				

